# Revolutionizing cancer immunotherapy in solid tumor: CAR engineering and single-cell sequencing insights

**DOI:** 10.3389/fimmu.2023.1310285

**Published:** 2023-11-23

**Authors:** Zuhui Pu, Tony Bowei Wang, Lisha Mou

**Affiliations:** ^1^ Imaging Department, Institute of Translational Medicine, The First Affiliated Hospital of Shenzhen University, Shenzhen Second People’s Hospital, Shenzhen, Guangdong, China; ^2^ MetaLife Lab, Shenzhen Institute of Translational Medicine, Shenzhen, Guangdong, China; ^3^ Biology Department, Skidmore College, Saratoga Springs, NY, United States

**Keywords:** CAR-T cell, CAR-M cell, CAR-NK cell, cancer immunotherapy, cellular immunotherapy, single-cell, scRNA-seq

## Abstract

The global increase in cancer incidence presents significant economic and societal challenges. While chimeric antigen receptor-modified T cell (CAR-T) therapy has demonstrated remarkable success in hematologic malignancies and has earned FDA approval, its translation to solid tumors encounters faces significant obstacles, primarily centered around identifying reliable tumor-associated antigens and navigating the complexities of the tumor microenvironment. Recent developments in single-cell RNA sequencing (scRNA-seq) have greatly enhanced our understanding of tumors by offering high-resolution, unbiased analysis of cellular heterogeneity and molecular patterns. These technologies have revolutionized our comprehension of tumor immunology and have led to notable progress in cancer immunotherapy. This mini-review explores the progress of chimeric antigen receptor (CAR) cell therapy in solid tumor treatment and the application of scRNA-seq at various stages following the administration of CAR cell products into the body. The advantages of scRNA-seq are poised to further advance the investigation of the biological characteristics of CAR cells in vivo, tumor immune evasion, the impact of different cellular components on clinical efficacy, the development of clinically relevant biomarkers, and the creation of new targeted drugs and combination therapy approaches. The integration of scRNA-seq with CAR therapy represents a promising avenue for future innovations in cancer immunotherapy. This synergy holds the potential to enhance the precision and efficacy of CAR cell therapies while expanding their applications to a broader range of malignancies.

## 1 Introduction

The escalating global cancer incidence presents substantial economic and societal burdens (1). Chimeric antigen receptor-modified T cell (CAR-T) therapy has achieved remarkable breakthroughs in treating hematologic malignancies. However, the extension of this success to solid tumors encounters formidable challenges, primarily centered around pinpointing reliable tumor-associated antigens and navigating the intricate complexities of the tumor microenvironment (TME) (2, 3). In recent years, single-cell RNA sequencing (scRNA-seq) has significantly improved our comprehension of tumors by enabling high-resolution, unbiased analysis of cellular heterogeneity and molecular patterns (4). These technologies provide unprecedented insights into these fields, which can result in significant advancements in cancer immunotherapy (5).

This review focuses on the progress of chimeric antigen receptor (CAR) cells in solid tumor therapy and the role of scRNA-seq in CAR cell therapy at various stages following the administration of CAR cell products into the body. Advancements in scRNA-seq will further promote the investigation of the biological characteristics of CAR cells *in vivo*, tumor immune evasion, the impact of different cellular components on clinical efficacy, the development of clinically relevant biomarkers, and the creation of new targeted drugs and combination therapy approaches. Ultimately, this review aims to enhance the clinical effectiveness of CAR cell therapy while minimizing any adverse reactions.

## 2 CAR-T, CAR-NK, CAR-M therapy

Adoptive cell therapy (ACT) employs patient-derived immune cells, particularly diverse T cell variants, which are cultured and genetically modified outside the body before being reintroduced to the patient as a therapeutic intervention for the identification and precise targeting of cancerous cells. Among the diverse ACT approaches, CAR-T therapy has gained prominence (6). CAR-T therapy for blood tumors has achieved remarkable remission rates of 80–90% and received FDA approval for clinical use. This success has spurred extensive research aimed at extending the benefits of CAR-T therapy to solid tumors (7).

However, CAR-T cell therapy faces numerous challenges and potential setbacks, including the advancement of illnesses in individuals, constraints in T cell collection, setbacks in CAR cell production, inadequate generation of CAR cells, inherent T cell deficiencies, and various additional factors. The TME plays a crucial role in CAR-T therapy (7). When CAR-T cells infiltrate the TME, they encounter a widespread immunosuppressive environment that hinders their functionality, particularly noticeable in specific cancers such as pancreatic cancer (8), Various cellular components within the TME, such as tumor-associated macrophages (TAM), regulatory T (Treg) cells, myeloid-derived suppressor cells (MDSC), and tumor angiogenesis factors (TAF), not only suppress CAR-T cell function but also contribute to the creation of an immune-suppressing cytokine environment and a metabolic microenvironment that weakens CAR-T cell activity. Key factors such as the vascular endothelial growth factor (VEGF) have been found to profoundly impact the immune system, inhibiting anti-cancer immune responses (9). Additionally, elements like TGF-β, IL-4, and IL-10 actively induce T cell dysfunction and support the entry of immunosuppressive immune cell populations (10). Various strategies have been developed and employed to enhance CAR-T cell therapy within the constraints of the TME. These strategies include integrating CAR-T cell therapy with immune checkpoint inhibitors (ICI) or alternative immunostimulatory treatments, along with modifying CAR-T cells to make them resistant to the impact of immunosuppressive cytokines (11).

Currently, multiple ongoing clinical trials are investigating the use of CAR-T cell therapy in the management of solid tumors, including lung cancer, liver cancer, and other tumor types (12–17). This immunotherapeutic strategy has shown considerable promise, yielding encouraging clinical results. Nevertheless, CAR-T cell treatment encounters numerous obstacles when targeting solid tumors, such as the absence of secure and efficient solid tumor antigens, inadequate movement and penetration of CAR-T cells into tumor locations, immunosuppressive TMEs, and antigen evasion during therapy.

**Figure 1:**
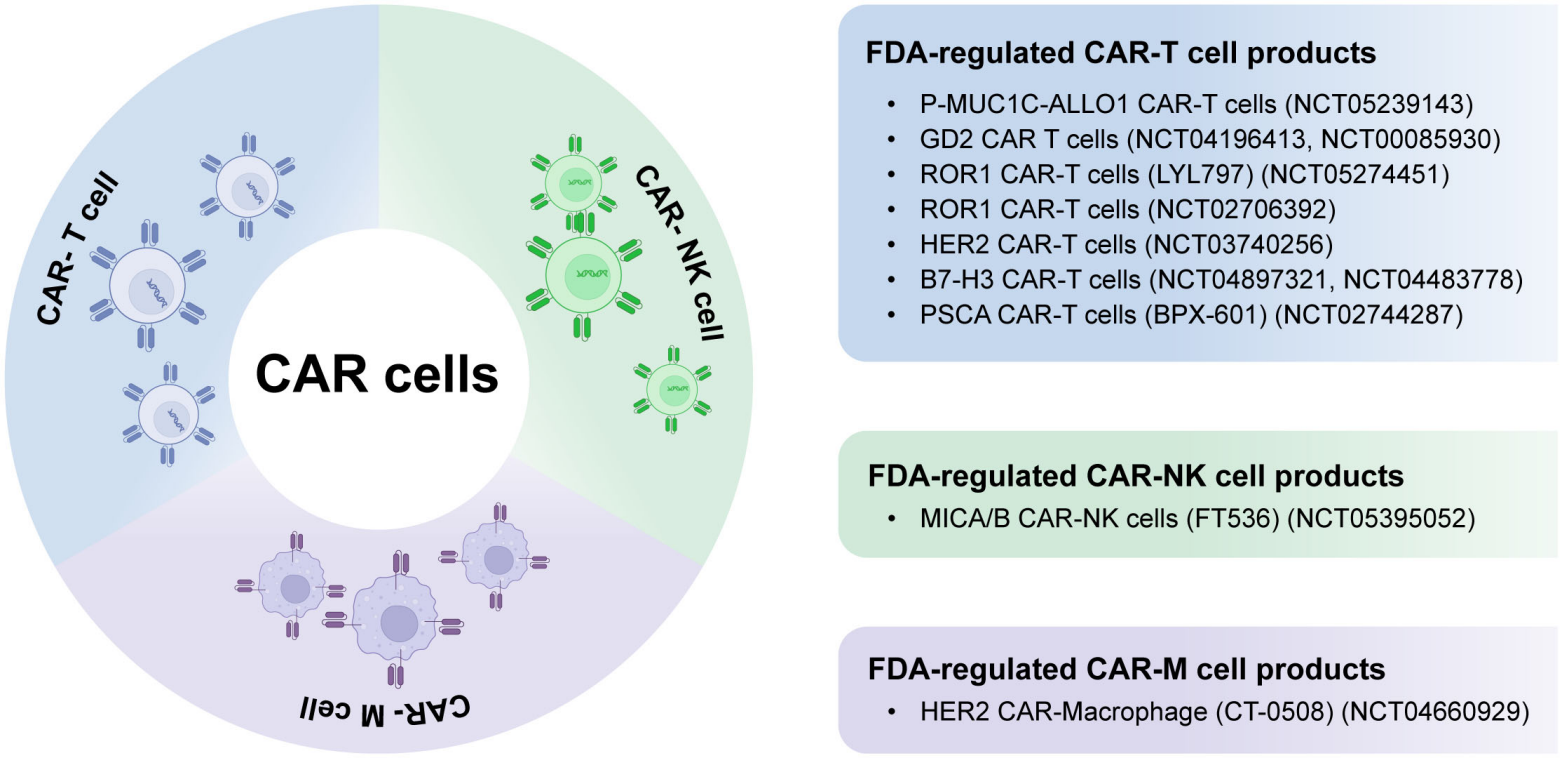
Summary of CAR-T, -NK, and –M in solid tumors treatment.

In recent times, CAR-natural killer (CAR-NK) and CAR-macrophage (CAR-M) therapies have gained increasing attention as potential supplements or alternatives to CAR-T cell therapy, specifically for addressing solid tumors (2, 6). **Figure 1** summarizes FDA-regulated CAR cell products, including CAR-T, CAR-NK, and CAR-M, in solid tumor treatment. CAR-NK cells are becoming a desirable alternative to CAR-T cells as they do not rely on human leukocyte antigen (HLA) compatibility, which mitigates concerns about toxicity. Additionally, CAR-NK cells have the potential to be produced in larger quantities from diverse origins, making them a highly promising ready-to-use solution for broader clinical implementation. On the contrary, CAR-M cell immunotherapy provides distinct functionalities like engulfment, presentation of tumor antigens, and profound infiltration into tumors (18–22).

CAR-NK cells offer unique advantages compared to CAR-T cells, as they can be generated from established cell lines or using allogeneic natural killer (NK) cells with different major histocompatibility complexes (MHC), broadening their range of applications. These cells can eliminate cancer cells using both CAR-mediated and CAR-independent methods while exhibiting reduced toxicity. CAR-NK cell therapy has been supported by clinical trials, demonstrating its efficacy and tolerability (23–28).

Macrophages, abundant within TMEs and highly skilled in penetrating tumors and regulating immune responses, present a promising avenue for CAR engineering. CAR-engineered macrophages have the potential to overcome various challenges associated with CAR-T and CAR-NK cell therapies, particularly in the context of solid tumors (6). However, it’s essential to recognize that both CAR-NK cells and CAR-M cells have their inherent limitations.

CAR-T cell therapy, while demonstrating remarkable success in hematological malignancies, faces considerable challenges when applied to solid tumors. These challenges arise from the unique microenvironment of solid tumors, characterized by immunosuppressive factors, limited T cell infiltration, and spatial heterogeneity. In order to address these challenges, several innovative strategies have emerged:

(1) Targeting the Tumor Microenvironment: One approach involves modifying CAR-T cells to secrete cytokines or factors that can remodel the TME, making it more conducive to immune cell activity.
(2) Enhancing T Cell Infiltration: Another strategy is to enhance T cell homing and infiltration into solid tumors include the engineering of CAR-T cells to include chemokine receptors specific to the TME.
(3) Combination Therapies: Finally, another possible strategy is the combination of CAR-T cell therapy with other immunotherapies or conventional treatments to create a synergistic effect.

In summary, the obstacles in solid tumor CAR-T cell therapy are indeed substantial, but ongoing research and innovative approaches are continuously advancing our ability to tackle these challenges effectively.

## 3 ScRNA-seq in CAR cell therapy

Single-cell RNA sequencing (scRNA-seq) is a powerful technique for deep sequencing of cellular mRNA, enabling high-throughput, quantitative profiling of gene expression and transcriptome signatures (29). However, traditional scRNA-seq methods often rely on the presence of established surface markers, potentially introducing bias into the analysis.

In the past, researchers generally considered cellular uniqueness and traits of a group to be identical. However, advances in oncological research have revealed significant heterogeneity among individual tumor cells, emphasizing the importance of single-cell analysis. Intra-tumor heterogeneity was first described in the 1800s by pathologist Rudolf Virchow (30). Due to technological limitations, researchers tried many ways to streamline the cell population, e.g., microarray/bulk sequencing after cell sorting, and a variety of algorithms were developed (31, 32). The development of sequencing technology makes scRNA-seq one of the most acceptable methods to date due to its ability to uncover distinguishing characteristics at the single-cell level (33–35). It allows for the creation of cell atlases, identification of distinct cellular subpopulations, examination of disease mechanisms, and exploration of potential therapeutic targets.

ScRNA-seq offers enhanced resolution of transcriptomes, providing a deeper understanding of cellular phenotypes (36). This technology has become crucial for studying immune cells, particularly in the context of immunotherapy scenarios (37, 38). ScRNA-seq has transformed the field by offering unbiased, detailed, and efficient sequencing analyses, providing an unprecedented view of cellular heterogeneity and intricate molecular landscapes. Functional investigations help understand cellular changes, connections, and communication systems involving CAR-T cells, offering fresh perspectives on the overall effectiveness of CAR-T cells, including its association with patient prognosis (39). We have summarized recent progress in applying scRNA-seq in CAR cell therapy in **Table 1** and CAR cell therapy targets analyzed by scRNA-seq in **Supplementary Table 1**.

**Table 1:** Main topics and key points of ScRNA-Seq in CAR cell therapy.

Main Topics and Key Points	Reference
1. ScRNA-Seq in CAR-T Cell Therapy: Efficacy, Development, and Mechanisms	
Long-term impact of CAR-T cell therapy in CLL	(40)
CD4+ CAR-T cell persistence and development	(40)
Gene expression patterns of CD19 CAR-T cells	(41)
Chimeric switch receptors (CARP) for enhanced activity	(41, 42)
Influence of co-stimulatory domains on engineered T cells	(43)
2. Guiding CAR-T Cell Development and Identifying Target Antigens	
Challenges of limited target antigens	(44)
Identification of potential CAR-T cell targets (CSF1R and CD86)	(44)
Design of memory-mimicking killer cells	(45)
Generation of diverse CAR variants with unique abilities	(44)
3. Understanding CAR-T Cell On-Target and Off-Tumor Effects, Antigen Escape, and Clonal Dynamics	
Insights into on-target and off-tumor effects	(46, 47)
Investigation of antigen expression patterns	(48)
Uncovering mechanisms of resistance	(49)
Analysis of clonal dynamics and transcriptional regulation	(50)
4. Revealing CAR-T Cell Interactions with the TME	
Study of interactions between CAR-T cell subclusters and the TME	(51)
Observation of changes in the TME during therapy	(51)
Exploration of CAR-T cell production of IFN-γ and its impact	(52)
Comparison of the roles of CD4+ and CD8+ CAR-T cells	(52)
5. Uncovering Mechanisms of Relapse in CAR-T Cell Therapy	
Investigation of reasons for relapse in CAR-T therapy	(53)
Examination of factors linked to relapse	(54)
Discovery of pre-existing subpopulations in patients	(55)
Potential solutions to prevent recurrence	(56)

### 3.1 ScRNA-seq in CAR-T therapy: efficacy, development, and mechanisms

ScRNA-seq has played a pivotal role in understanding the efficacy, development, and mechanisms of CAR-T cell therapy. Long-term studies by Melenhorst et al. have demonstrated the enduring impact of CAR-T cell therapy in treating chronic lymphocytic leukemia (CLL), particularly in tracking the persistence and development of CD4+ CAR-T cells over a decade (40). Furthermore, gene expression patterns of CD19 CAR-T cells in relapsed/refractory childhood pre-B cell acute lymphoblastic leukemia (R/R B-ALL) patients have revealed valuable insights into the memory-like state and the use of chimeric switch receptors (CARP) for enhancing anti-cancer activity (41, 42). Boroughs et al. have provided an in-depth analysis of how different co-stimulatory domains shape the transcriptional programs of engineered T cells, offering practical guidance for CAR design (43).

### 3.2 Guiding CAR-T development and identifying target antigens

ScRNA-seq has played a critical role in addressing the ongoing issue of scarce safe target antigens for CAR-T cell therapy. Analysis of more than 500,000 cells, including those from acute myeloid leukemia (AML) patients and healthy controls, has led to the identification of potential CAR-T cell targets like CSF1R and CD86 (44). These targets have demonstrated robust efficacy while minimizing off-target toxicity, providing a compelling rationale for clinical development. Additionally, studies have leveraged scRNA-seq to design memory-mimicking killer cells and “speedingCars” for CAR-T cell development (45). By utilizing natural signaling domains, researchers have generated 180 CAR variants with unique abilities to eliminate tumors and exhibit distinct T cell characteristics, expanding the possibilities for therapeutic CAR engineering (44).

### 3.3 Understanding CAR-T cell on-target and off-tumor effects, antigen escape, and clonal dynamics

ScRNA-seq has been a vital tool for comprehending on-target and off-tumor effects in CAR-T cell therapy (46, 47). Through scRNA-seq, scientists have gained insights into the expression patterns of CAR targets in various tissues, providing valuable knowledge about the mechanisms behind on-target and off-tumor toxicity (48). This information can guide the implementation of preventive measures during treatment. Additionally, studies have explored antigen expression patterns in both standard and malignant cells, uncovering the factors driving resistance in CAR therapies targeting BCMA and GPRC5D in multiple myeloma (49). ScRNA-seq has also unveiled clonal dynamics and transcriptional regulation post-infusion of CAR-T cells, offering a deeper understanding of treatment responses at the molecular level (50).

### 3.4 ScRNA-seq revealed mechanism for neurotoxicity in CAR-T cell therapy

The interaction between CAR-T cell subclusters and the TME is crucial for the success of CAR-T cell therapy, with scRNA-seq being an instrumental part of studying this dynamic interaction. Using a mouse model of B cell lymphoma treated with anti-CD19 CAR-T cell therapy, significant changes in the TME during therapy were observed (51). CAR-T cells produced IFN-γ, which enhanced the activity of host T and natural killer cells while sustaining CAR-T cell cytotoxicity. The ability of CD4+ CAR-T cells to activate the host immune response and the effectiveness of CD8+ CAR-T cells in directly eliminating tumors have been highlighted (52). These findings underscore the importance of CAR-T cell interactions with the TME and suggest that enhancing these interactions could help prevent therapy relapses.

### 3.5 Uncovering mechanisms of relapse in CAR-T cell therapy

Relapses are a common challenge in CAR-T cell therapy, both in cases of CD19-positive and CD19-negative disease. However, scRNA-Seq has been a valuable tool in uncovering the mechanisms behind relapse. By examining single-cell data obtained from infusion products, researchers have identified factors linked to relapse, including a lack of TH2 activity in CD19-positive cases (53). TIGIT expression, detected by scRNA-seq, can lead CAR-T cells into a non-proliferative and exhausted state, especially in poorly responsive patients. Inhibiting TIGIT holds promise for enhancing CAR-T cell anti-tumor function (54). Variations in pre-manufactured T cell features significantly impact CAR-T therapy efficacy. Loss of CCR7 gene expression and altered gene expression in CD8+ naïve-T cell populations, as identified by scRNA-seq, are associated with poor molecular responses (55). ScRNA-seq has also been used to demonstrate the existence of pre-existing subpopulations in patients, providing guidance for treatment approaches aimed at preventing recurrence (56). This section explores mechanisms and potential solutions to prevent relapse in CAR-T cell therapy, including TIGIT-related dysfunction and the impact of pre-manufactured T cell features.

## 4 Future directions in CAR cell therapy and scRNA-seq

While this mini-review has aimed to provide an overview of the current applications of single-cell RNA sequencing (scRNA-seq) in CAR therapy, it is essential to consider the potential future directions in CAR cell therapy and how scRNA-seq might contribute to these developments.

### 4.1 Future directions in CAR cell therapy

Looking ahead, CAR cell therapy holds promise in several key areas:

(1) Personalized Medicine: The integration of scRNA-seq data with patient-specific genomic information can lead to the development of personalized CAR therapies, tailored to the unique molecular characteristics of a patient’s tumor.
(2) Next-Generation CARs: The engineering of novel CAR constructs, such as universal CAR-T cells, bi-specific CARs, or armored CARs, will expand the applicability of CAR therapies to a broader spectrum of cancers.
(3) Reducing Toxicity: Efforts to mitigate the side effects and cytokine release syndrome associated with CAR-T cell therapy are ongoing, and future therapies are likely to be safer and more targeted.

### 4.2 The role of scRNA-seq in advancing CAR therapy

As we envision the future of CAR cell therapy, scRNA-seq will continue to play a pivotal role:

(1) Mapping Tumor Heterogeneity: ScRNA-seq enables in-depth characterization of the heterogeneous cell populations within a tumor, aiding in the identification of new target antigens and the development of more effective CAR constructs.
(2) Tracking CAR-T Cells: ScRNA-seq can be employed to monitor CAR-T cell behavior within the patient, helping to optimize dosing, predict responses, and evaluate long-term effects.
(3) Understanding Resistance Mechanisms: By analyzing single-cell transcriptomes, we can uncover the resistance mechanisms that solid tumors may employ against CAR-T cell therapy, thereby guiding the development of strategies to overcome them.

By combining scRNA-seq with CAR therapy, it offers an exciting avenue for future innovations in cancer immunotherapy. This synergy holds the potential to enhance the precision and efficacy of CAR cell therapies while expanding their applications to a broader range of malignancies.

## 5 Comment

Despite certain limitations, such as the challenge of accurately linking gene transcription to protein expression, scRNA-seq is increasingly recognized for its potential in CAR-T cell therapy. ScRNA-seq has been invaluable for investigating the molecular complexities and diversity of cell populations engineered with a CAR design. Nevertheless, the potential of scRNA-seq to analyze intricate cell populations suggests an exciting opportunity for concurrent screening of diverse CAR constructs. This approach could enable the association of specific CAR cell transcriptomic profiles with optimal applications or guide the personalized design of CARs tailored to individual patients’ distinct requirements, thereby enhancing the potential of immunotherapy as a form of personalized healthcare.

## Author contributions

ZP: Conceptualization, Writing – review & editing. TW: Writing – original draft. LM: Conceptualization, Writing – original draft, Writing – review & editing.
