# Supplementary material for: Revolutionizing cancer immunotherapy in solid tumor: CAR engineering and single-cell sequencing insights
Source: Front Immunol. 2023 Nov 23;14:1310285. doi: 10.3389/fimmu.2023.1310285 (PMC10712310; doi:10.3389/fimmu.2023.1310285)
Supplement: Supplementary file 1 [file Table_1.docx]

| **Supplementary Table 1. Summary of CAR Cell Therapy Targets Analyzed by ScRNA-Seq.** | | | |
| --- | --- | --- | --- |
| **Targets** | **Technology** | **Tumor type** | **Citation** |
| **CD19** | | | |
|  | scRNA-seq | B-ALL, BCL, CNSL | (1–4) |
|  | scRNA-seq, flow cytometry | B-ALL | (5) |
|  | scRNA-seq, flow cytometry | NHL | (6) |
|  | scRNA-seq, CITE-seq, scTCR-seq, CyTOF | CLL | (7) |
|  | scRNA-seq, CITE-seq, flow cytometry | ALL | (8) |
|  | scRNA-seq, CRISPR/Cas9 genome editing system | B-NHL | (9) |
|  | scRNA-seq, image analysis, flow cytometry | BCL | (10) |
|  | scRNA-seq, scTCR-seq | B-ALL | (11) |
|  | scRNA-seq, scTCR-seq flow cytometry | LBCL | (12) |
|  | scRNA-seq, scTCR-seq, cytokine multiplex profiling | MCL | (13) |
|  | scRNA-seq, bulk RNA-seq | B-ALL | (14) |
|  | scRNA-seq, bulk RNA-seq, flow cytometry | B-ALL | (15) |
|  | scRNA-seq, bulk RNA-seq, flow cytometry | DLBCL | (16) |
|  | scRNA-seq, CITE-seq | ALL | (17) |
|  | scRNA-seq, cytokine profiling, flow cytometry | NHL | (18) |
|  | scRNA-seq, scTCR-seq, CITE-seq, CyTOF | LBCL | (19) |
|  | scRNA-seq, single-cell cytokine assay | BCL | (20) |
|  | scRNA-seq, TCR-seq | CLL, NHL | (21) |
| **BCMA** |  |  |  |
|  | scRNA-seq, flow cytometry | PCL | (22) |
|  | scRNA-seq, flow cytometry | MM | (23) |
|  | scRNA-seq, scTCR-seq, flow cytometry, CITE-seq | MM | (24) |
|  | scRNA-seq, ATAC-seq, flow cytometry | MM | (25) |
| **CD19, CD44v6** | | | |
|  | scRNA-seq | B-ALL | (26) |
| **CD19, MSLN** | | | |
|  | scRNA-seq | MM, PAAD | (27) |
| **GD2** | | | |
|  | scRNA-seq, CRISPR/Cas9 genome editing system | NB | (28) |
| **Her2** | | | |
|  | scRNA-seq, flow cytometry | BRCA | (29) |
| **IL13Rα2** | | | |
|  | scRNA-seq, genome-wide CRISPR/Cas9 knockout screening | GBM | (30) |
| **NPM1c** | | | |
|  | scRNA-seq, flow cytometry | AML | (31) |
| **TRP1** |  |  |  |
|  | scRNA-seq | MM | (32) |
|  | | | |
|  |  |  |  |
|  | | | |

**Reference**

1. Wang X, Huynh C, Urak R, Weng L, Walter M, Lim L, Vyas V, Chang W-C, Aguilar B, Brito A, et al. The Cerebroventricular Environment Modifies CAR T Cells for Potent Activity against Both Central Nervous System and Systemic Lymphoma. *Cancer Immunol Res* (2021) 9:75–88. doi: 10.1158/2326-6066.CIR-20-0236

2. Parker KR, Migliorini D, Perkey E, Yost KE, Bhaduri A, Bagga P, Haris M, Wilson NE, Liu F, Gabunia K, et al. Single-Cell Analyses Identify Brain Mural Cells Expressing CD19 as Potential Off-Tumor Targets for CAR-T Immunotherapies. *Cell* (2020) 183:126-142.e17. doi: 10.1016/j.cell.2020.08.022

3. Im NG, Guillaumet-Adkins A, Wal M, Rogers AJ, Frede J, Havig CC, Yang J, Anand P, Stegmann SK, Waldschmidt JM, et al. Regulatory Programs of B-cell Activation and Germinal Center Reaction Allow B-ALL Escape from CD19 CAR T-cell Therapy. *Cancer Immunol Res* (2022) 10:1055–1068. doi: 10.1158/2326-6066.CIR-21-0626

4. Rabilloud T, Potier D, Pankaew S, Nozais M, Loosveld M, Payet-Bornet D. Single-cell profiling identifies pre-existing CD19-negative subclones in a B-ALL patient with CD19-negative relapse after CAR-T therapy. *Nat Commun* (2021) 12:865. doi: 10.1038/s41467-021-21168-6

5. Anderson ND, Birch J, Accogli T, Criado I, Khabirova E, Parks C, Wood Y, Young MD, Porter T, Richardson R, et al. Transcriptional signatures associated with persisting CD19 CAR-T cells in children with leukemia. *Nat Med* (2023) 29:1700–1709. doi: 10.1038/s41591-023-02415-3

6. Jackson Z, Hong C, Schauner R, Dropulic B, Caimi PF, de Lima M, Giraudo MF, Gupta K, Reese JS, Hwang TH, et al. Sequential single cell transcriptional and protein marker profiling reveals TIGIT as a marker of CD19 CAR-T cell dysfunction in patients with non-Hodgkin’s lymphoma. *Cancer Discov* (2022) 12:1886–1903. doi: 10.1158/2159-8290.CD-21-1586

7. Melenhorst JJ, Chen GM, Wang M, Porter DL, Chen C, Collins MA, Gao P, Bandyopadhyay S, Sun H, Zhao Z, et al. Decade-long leukaemia remissions with persistence of CD4+ CAR T cells. *Nature* (2022) 602:503–509. doi: 10.1038/s41586-021-04390-6

8. Bai Z, Woodhouse S, Zhao Z, Arya R, Govek K, Kim D, Lundh S, Baysoy A, Sun H, Deng Y, et al. Single-cell antigen-specific landscape of CAR T infusion product identifies determinants of CD19-positive relapse in patients with ALL. *Sci Adv* (2022) 8:eabj2820. doi: 10.1126/sciadv.abj2820

9. Zhang J, Hu Y, Yang J, Li W, Zhang M, Wang Q, Zhang L, Wei G, Tian Y, Zhao K, et al. Non-viral, specifically targeted CAR-T cells achieve high safety and efficacy in B-NHL. *Nature* (2022) 609:369–374. doi: 10.1038/s41586-022-05140-y

10. Boulch M, Cazaux M, Loe-Mie Y, Thibaut R, Corre B, Lemaître F, Grandjean CL, Garcia Z, Bousso P. A cross-talk between CAR T cell subsets and the tumor microenvironment is essential for sustained cytotoxic activity. *Sci Immunol* (2021) 6:eabd4344. doi: 10.1126/sciimmunol.abd4344

11. Wilson TL, Kim H, Chou C-H, Langfitt D, Mettelman RC, Minervina AA, Allen EK, Métais J-Y, Pogorelyy MV, Riberdy JM, et al. Common Trajectories of Highly Effective CD19-Specific CAR T Cells Identified by Endogenous T-cell Receptor Lineages. *Cancer Discov* (2022) 12:2098–2119. doi: 10.1158/2159-8290.CD-21-1508

12. Haradhvala NJ, Leick MB, Maurer K, Gohil SH, Larson RC, Yao N, Gallagher KME, Katsis K, Frigault MJ, Southard J, et al. Distinct cellular dynamics associated with response to CAR-T therapy for refractory B cell lymphoma. *Nat Med* (2022) 28:1848–1859. doi: 10.1038/s41591-022-01959-0

13. Jiang VC, Hao D, Jain P, Li Y, Cai Q, Yao Y, Nie L, Liu Y, Jin J, Wang W, et al. TIGIT is the central player in T-cell suppression associated with CAR T-cell relapse in mantle cell lymphoma. *Mol Cancer* (2022) 21:185. doi: 10.1186/s12943-022-01655-0

14. Boroughs AC, Larson RC, Marjanovic ND, Gosik K, Castano AP, Porter CBM, Lorrey SJ, Ashenberg O, Jerby L, Hofree M, et al. A Distinct Transcriptional Program in Human CAR T Cells Bearing the 4-1BB Signaling Domain Revealed by scRNA-Seq. *Mol Ther* (2020) 28:2577–2592. doi: 10.1016/j.ymthe.2020.07.023

15. Bueno C, Barrera S, Bataller A, Ortiz-Maldonado V, Elliot N, O’Byrne S, Wang G, Rovira M, Gutierrez-Agüera F, Trincado JL, et al. CD34+CD19-CD22+ B-cell progenitors may underlie phenotypic escape in patients treated with CD19-directed therapies. *Blood* (2022) 140:38–44. doi: 10.1182/blood.2021014840

16. Wang Y, Tong C, Lu Y, Wu Z, Guo Y, Liu Y, Wei J, Wang C, Yang Q, Han W. Characteristics of premanufacture CD8+T cells determine CAR-T efficacy in patients with diffuse large B-cell lymphoma. *Signal Transduct Target Ther* (2023) 8:409. doi: 10.1038/s41392-023-01659-2

17. Bai Z, Lundh S, Kim D, Woodhouse S, Barrett DM, Myers RM, Grupp SA, Maus MV, June CH, Camara PG, et al. Single-cell multiomics dissection of basal and antigen-specific activation states of CD19-targeted CAR T cells. *J Immunother Cancer* (2021) 9:e002328. doi: 10.1136/jitc-2020-002328

18. Rossi J, Paczkowski P, Shen Y-W, Morse K, Flynn B, Kaiser A, Ng C, Gallatin K, Cain T, Fan R, et al. Preinfusion polyfunctional anti-CD19 chimeric antigen receptor T cells are associated with clinical outcomes in NHL. *Blood* (2018) 132:804–814. doi: 10.1182/blood-2018-01-828343

19. Good Z, Spiegel JY, Sahaf B, Malipatlolla MB, Ehlinger ZJ, Kurra S, Desai MH, Reynolds WD, Wong Lin A, Vandris P, et al. Post-infusion CAR T(Reg) cells identify patients resistant to CD19-CAR therapy. *Nat Med* (2022) 28:1860–1871. doi: 10.1038/s41591-022-01960-7

20. Xhangolli I, Dura B, Lee G, Kim D, Xiao Y, Fan R. Single-cell Analysis of CAR-T Cell Activation Reveals A Mixed TH1/TH2 Response Independent of Differentiation. *Genomics Proteomics Bioinformatics* (2019) 17:129–139. doi: 10.1016/j.gpb.2019.03.002

21. Sheih A, Voillet V, Hanafi L-A, DeBerg HA, Yajima M, Hawkins R, Gersuk V, Riddell SR, Maloney DG, Wohlfahrt ME, et al. Clonal kinetics and single-cell transcriptional profiling of CAR-T cells in patients undergoing CD19 CAR-T immunotherapy. *Nat Commun* (2020) 11:219. doi: 10.1038/s41467-019-13880-1

22. Li X, Guo X, Zhu Y, Wei G, Zhang Y, Li X, Xu H, Cui J, Wu W, He J, et al. Single-Cell Transcriptomic Analysis Reveals BCMA CAR-T Cell Dynamics in a Patient with Refractory Primary Plasma Cell Leukemia. *Mol Ther* (2021) 29:645–657. doi: 10.1016/j.ymthe.2020.11.028

23. Lee H, Ahn S, Maity R, Leblay N, Ziccheddu B, Truger M, Chojnacka M, Cirrincione A, Durante M, Tilmont R, et al. Mechanisms of antigen escape from BCMA- or GPRC5D-targeted immunotherapies in multiple myeloma. *Nat Med* (2023) 29:2295–2306. doi: 10.1038/s41591-023-02491-5

24. Dhodapkar KM, Cohen AD, Kaushal A, Garfall AL, Manalo RJ, Carr AR, McCachren SS, Stadtmauer EA, Lacey SF, Melenhorst JJ, et al. Changes in Bone Marrow Tumor and Immune Cells Correlate with Durability of Remissions Following BCMA CAR T Therapy in Myeloma. *Blood Cancer Discov* (2022) 3:490–501. doi: 10.1158/2643-3230.BCD-22-0018

25. Rodriguez-Marquez P, Calleja-Cervantes ME, Serrano G, Oliver-Caldes A, Palacios-Berraquero ML, Martin-Mallo A, Calviño C, Español-Rego M, Ceballos C, Lozano T, et al. CAR density influences antitumoral efficacy of BCMA CAR T cells and correlates with clinical outcome. *Sci Adv* (2022) 8:eabo0514. doi: 10.1126/sciadv.abo0514

26. Norelli M, Camisa B, Barbiera G, Falcone L, Purevdorj A, Genua M, Sanvito F, Ponzoni M, Doglioni C, Cristofori P, et al. Monocyte-derived IL-1 and IL-6 are differentially required for cytokine-release syndrome and neurotoxicity due to CAR T cells. *Nat Med* (2018) 24:739–748. doi: 10.1038/s41591-018-0036-4

27. Johnson LR, Lee DY, Eacret JS, Ye D, June CH, Minn AJ. The immunostimulatory RNA RN7SL1 enables CAR-T cells to enhance autonomous and endogenous immune function. *Cell* (2021) 184:4981-4995.e14. doi: 10.1016/j.cell.2021.08.004

28. Mueller KP, Piscopo NJ, Forsberg MH, Saraspe LA, Das A, Russell B, Smerchansky M, Cappabianca D, Shi L, Shankar K, et al. Production and characterization of virus-free, CRISPR-CAR T cells capable of inducing solid tumor regression. *J Immunother Cancer* (2022) 10:e004446. doi: 10.1136/jitc-2021-004446

29. Xu N, Palmer DC, Robeson AC, Shou P, Bommiasamy H, Laurie SJ, Willis C, Dotti G, Vincent BG, Restifo NP, et al. STING agonist promotes CAR T cell trafficking and persistence in breast cancer. *J Exp Med* (2021) 218:e20200844. doi: 10.1084/jem.20200844

30. Wang D, Prager BC, Gimple RC, Aguilar B, Alizadeh D, Tang H, Lv D, Starr R, Brito A, Wu Q, et al. CRISPR Screening of CAR T Cells and Cancer Stem Cells Reveals Critical Dependencies for Cell-Based Therapies. *Cancer Discov* (2021) 11:1192–1211. doi: 10.1158/2159-8290.CD-20-1243

31. Dong H, Ham JD, Hu G, Xie G, Vergara J, Liang Y, Ali A, Tarannum M, Donner H, Baginska J, et al. Memory-like NK cells armed with a neoepitope-specific CAR exhibit potent activity against NPM1 mutated acute myeloid leukemia. *Proc Natl Acad Sci U S A* (2022) 119:e2122379119. doi: 10.1073/pnas.2122379119

32. Brog RA, Ferry SL, Schiebout CT, Messier CM, Cook WJ, Abdullah L, Zou J, Kumar P, Sentman CL, Frost HR, et al. Superkine IL-2 and IL-33 Armored CAR T Cells Reshape the Tumor Microenvironment and Reduce Growth of Multiple Solid Tumors. *Cancer Immunol Res* (2022) 10:962–977. doi: 10.1158/2326-6066.CIR-21-0536
